# First draft genome sequence of the rock bream in the family *Oplegnathidae*

**DOI:** 10.1038/sdata.2018.234

**Published:** 2018-10-23

**Authors:** Younhee Shin, Myunghee Jung, Ga-hee Shin, Ho-jin Jung, Su-Jin Baek, Gi-Yong Lee, Byeong-Chul Kang, Jaeyoung Shim, Ji-man Hong, Jung Youn Park, Cheul Min An, Young-Ok Kim, Jae Koo Noh, Ju-Won Kim, Bo-Hye Nam, Chan-Il Park

**Affiliations:** 1 Research and Development Center, Insilicogen Inc., Yongin-si 16954, Gyeonggi-do, Republic of Korea; 2 Department of Biological Sciences, Sungkyunkwan University, Suwon, 16419, Republic of Korea; 3 Department of Forest Science, Research Institute of Agriculture and Life Science, College of Agriculture and Life Sciences, Seoul National University, Seoul, 08826, Republic of Korea; 4 Biotechnology Research Division, National Institute of Fisheries Science, Haean-ro 216, Gijang-eup, Gijang-gun, Busan, 46083, Republic of Korea; 5 Department of Marine Biology & Aquaculture, College of Marine Science, Gyeongsang National University, 455, Tongyeong, 53064, Republic of Korea

**Keywords:** Genomics, DNA, DNA sequencing

## Abstract

The rock bream (*Oplegnathus fasciatus*) is one of the most economically valuable marine fish in East Asia, and due to various environmental factors, there is substantial revenue loss in the production sector. Therefore, knowledge of its genome is required to uncover the genetic factors and the solutions to these problems. In this study, we constructed the first draft genome of *O. fasciatus* as a reference for the family *Oplegnathidae*. The genome size is estimated to be 749 Mb, and it was assembled into 766 Mb by combining Illumina and PacBio sequences. A total of 24,053 transcripts (23,338 genes) are predicted, and among those transcripts, 23,362 (97%), are annotated with functional terms. Finally, the completeness of the genome assembly was assessed by CEGMA, which resulted in the complete mapping of 220 (88.7%) core genes in the genome. To the best of our knowledge, this is the first draft genome for the family *Oplegnathidae*.

## Background & Summary

*Oplegnathus fasciatus* (commonly known as rock bream, barred knifejaw or striped beakfish), is a fish belonging to the family *Oplegnathidae*. Those common names are derived from its phenotypic features. Rock bream is a subtropical and carnivorous species and is an economically important teleost fish in East Asia^1^. Generally, the rock bream inhabits estuaries at various depths according to their growth stage, i.e., as juveniles, they are mostly found in drifting seaweed/algae, and as adults, they are present at depths of 1 to 10 meters^1^. Moreover, the species growth depends on the photoperiod^2^. Other factors, such as overfishing and environmental changes, are affecting fish yield and cost, particularly in wild conditions. To overcome these issues, *O. fasciatus* is propagated via aquaculture to achieve sustainable and cost-effective production. In 2008, the annual production of *O. fasciatus* in South Korea was 614 tons, and that figure had increased to 909 tons in 2016^3^. However, bacterial and viral diseases cause an enormous economic loss in the Korean aquaculture industry^3^. As a consequence, the scientific community continues to seek various solutions, including molecular genetic applications, to overcome those problems. Some examples of these applications include genetic breeding^4^, QTL marker identification^5^, characterization of immunological pathway genes, proposed sex determination^6^, sex chromosomal evolution models^6^, antimicrobial peptides^7,8^, and vaccine development^9^.

**Figure 1:**
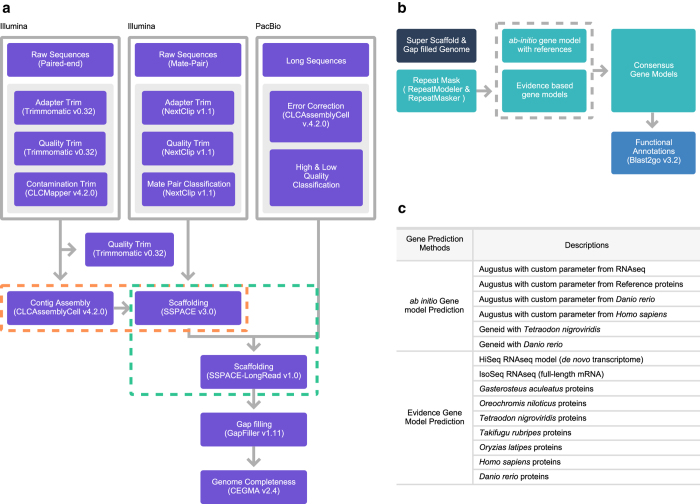
Illustration of the complete *Oplegnathus fasciatus* genome assembly and the structural and functional annotation pipelines used. (**a**) the genome assembly pipeline, (**b**) the structural and functional annotation pipeline, (**c**) details of the reference gene sets used for the *ab initio* and evidence-based gene model predictions.

More and more often, advances in molecular sequencing technologies are supporting the scientific community in uncovering the inherited molecular mechanisms of a given species, rather than depending on its model organism^10^. In this study, we constructed a draft genome for *O. fasciatus* using next-generation sequencing (NGS) (Fig. 1), which could aid in functional characterization of *O. fasciatus*-associated problems.

**Figure 2:**
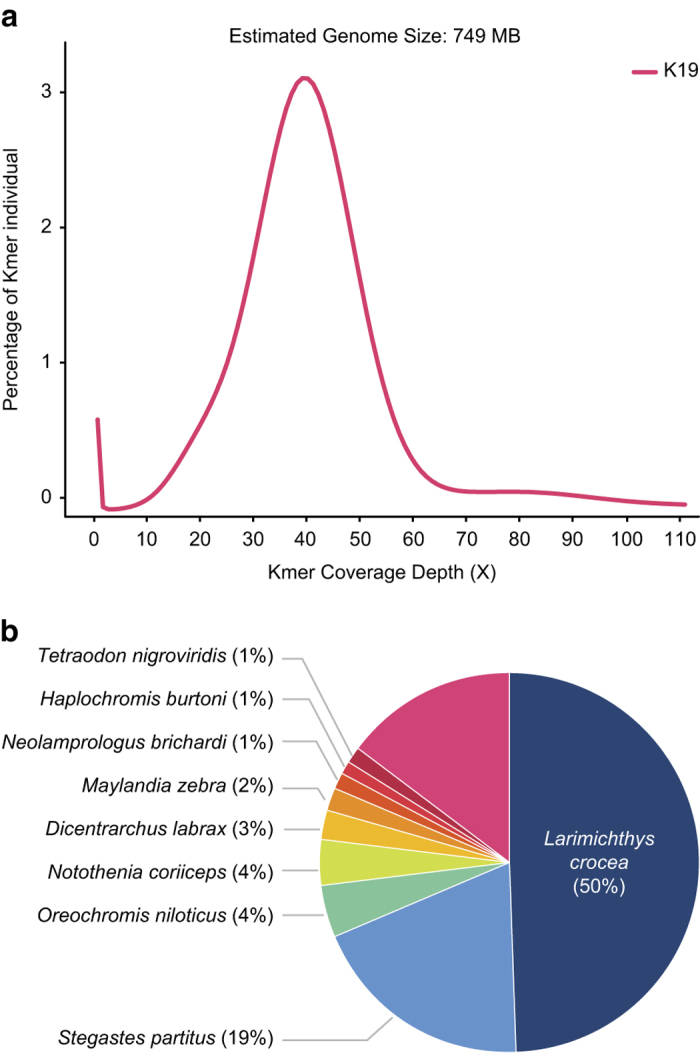
Illustration of the genome size and the functional annotation of the *Oplegnathus fasciatus* genome. (**a**) *k*-mer based genome size estimation, (**b**) sequence similarity-based species distribution obtained from BLAST.

The *O. fasciatus* genome size is estimated to be ~749 Mb (Fig. 2a) and was assembled into scaffolds with a total size of 762 Mb. Initially, the 224 Gb Illumina library (Table 1) assembled into 108,639 contigs and 31,533 scaffolds. Although the assembled scaffolds are larger than the estimated genome size, it is highly fragmented (Table 2). Therefore, the inclusion of 11.5 Gb of PacBio sequences in the second assembly improved the quality of the overall draft genome when compared to the initial assembly (Fig. 1a). This addition resulted in a 766 Mb draft genome with 4,149 scaffolds, along with improvements to the N50 (0.87 Mb to 1.1 Mb) and to the gaps (5.3% to 5.2%) (Table 2). Furthermore, the repeats were predicted by the *de novo* method were classified into subclasses (Table 3). In total, 180 Mb (23.56%) of genomic regions consist of repeat sequences, and it is masked in the genome.

A total of 334.3 Gb of mRNA transcriptome sequences from 34 libraries (313.8 Gb of Illumina data and 20.5 Gb of Iso-Seq data) was used for the EVM, and seven genomes were used in the *ab initio* gene modeler. These analyses predicted 23,338 genes and 24,053 transcripts, and 23,362 (97%) of those transcripts were annotated from biological databases. Moreover, the completeness score produced from CEGMA indicated that 220 (88.7%) eukaryotic core genes are entirely mapped to the genome. Therefore, these results clearly show that the given draft genome could be a near-complete reference genome for *O. fasciatus*. Moreover, these scaffolds will act as a primary genetic resource for *O. fasciatus* that can be used to design functional studies, and the annotated transcripts (97%) will aid in detailed characterizations. Finally, based on a literature survey and author knowledge, this is the first draft genome presented to the public from the family of *Oplegnathidae*; therefore, these data could be a valuable asset for marine researchers.

## Methods

### Sample collection and genomic DNA extraction

A single rock bream fish (95 ± 5 g) was supplied by the Gyeongsangnam-do Fisheries Resources Research Institute (FRRI) (Tongyeong, Republic of Korea) and was maintained at 22 ± 0.5 °C in aerated seawater. Liver tissue was taken from the fresh rock bream aseptically and stored in liquid nitrogen for the extraction of the genomic DNA. The genomic DNA was extracted using a DNeasy Animal Mini Kit (Qiagen, Hilden, Germany). A total of 24 μg of DNA was quantified using the standard procedure for the Quant-iT PicoGreen ds-DNA Assay Kit (Molecular Probes, Eugene, OR, USA) with a Synergy HTX Multi-Mode Reader (Biotek, Winooski, VT, USA). The quality of the DNA was also checked using an ND-1000 spectrophotometer (Thermo Scientific, Wilmington, DE, USA).

### DNA library preparation and sequencing

High-quality high molecular-weight genomic DNA > 100 kb in length was isolated from the given tissues, and two protocols were used to construct the sequencing libraries according to the manufacturer protocols, i.e., Illumina paired-end (PE) and mate pair (MP) libraries, (Illumina, San Diego, CA, USA). Furthermore, these libraries were fragmented and size-selected for Illumina Hi-Seq sequencing (Table 1). To obtain long non-fragmented sequence reads from the libraries, the PacBio manufacturing protocols were used (Pacific Biosciences, CA, USA) with 14 cells, and the sequencing used the P6-C4 chemistry of the PacBio RS II system (Table 1).

### Preprocessing and genome size estimation

The entire Illumina DNA sequences were subjected to pre-processing steps, which included adapter trimming, quality trimming (Q20) and contamination removal. The adapter and quality trims were conducted by using Trimmomatic-0.32 functions^11^, and the microbial contamination of each sample was removed by CLCMapper v4.2.0 (https://www.qiagenbioinformatics.com/products/clc-assembly-cell/) with an in-house database. Here, the in-house database was constructed from the meta-genomes (bacteria (ftp://ftp.ncbi.nlm.nih.gov/genomes/GENOME_REPORTS/prokaryotes.txt), virus (ftp://ftp.ncbi.nlm.nih.gov/genomes/Viruses/) and marine metagenomes (https://www.ncbi.nlm.nih.gov/bioproject/PRJNA13694). Similarly, mate pair sequences were also subjected to adapter and quality trimming, and classification of the mate pairs was performed using the Nextclip v1.1 method^12^. All the pre-processed sequences (Insert size: 550 bp, 35 Gb) from the paired-end library (Data Citation 1) were subjected to genome size estimation using the *k*-mer based method (which was used in the panda genome^13^). The *k*-mer frequencies (*k*-mer size = 19) were obtained using the Jellyfish v2.0 method^14^, and the genome size was calculated from the given formulas: Genome Coverage Depth = (*k*-mer Coverage Depth × Average Read Length)/(Average Read Length – *k*-mer size + 1) and Genome size = Total Base Number/Genome Coverage Depth. Alternatively, the PacBio sequences were only subjected to error correction using CLCAssemblyCell v4.2.0 (Fig. 1a).

### *De novo* Genome Assembly and Scaffolds

The draft genome was built from two type of assemblies, i.e., short-read assemblies and hybrid assemblies. Initially, the complete pre-processed paired-end DNA sequences were subjected to CLCAssemblyCell v4.2.0 to build the contigs. Furthermore, it was scaffolded with mate-pair sequences using the SSPACE v3.0 method^15^, and the hybrid assembly was built with the SSPACE-LongRead v1.0 method^16^ from the scaffolds along with the processed PacBio sequences. Next, the hybrid scaffolds were subjected to gap filling with paired-end and mate pair libraries using the GapFiller 1.11 method^17^. Finally, the gene completeness was assessed using CEGMA^18^ (Fig. 1a).

### *De novo* repeat region prediction and classification

Initially, repeat regions were predicted using the *de novo* method and classified into repeat subclasses (Table 3). The *de novo* repeat prediction for *O. fasciatus* was conducted using RepeatModeler (http://www.repeatmasker.org/RepeatModeler/), which includes other methods such as RECON^19^ (http://eddylab.org/software/recon/), RepeatScout^20^ (https://bix.ucsd.edu/repeatscout/) and TRF^21^ (https://tandem.bu.edu/trf/trf.html). Furthermore, the repeats were masked using RepeatMasker v4.0.5 (http://www.repeatmasker.org/) with RMBlastn v2.2.27^+^ and classified into their subclasses using the Repbase^22^ v20.08 databases for reference (https://www.girinst.org/repbase/).

### Gene prediction and annotation

The genes from the *O. fasciatus* draft genome were predicted using an in-house gene prediction pipeline, which includes three modules: an evidence-based gene modeler (EVM), an *ab initio* gene modeler and a consensus gene modeler. Finally, the functional annotation processing was conducted for the consensus genes (Fig. 1b). The details of this pipeline were previously explained in articles on the genomes of *Capsicum*^23^ and *Haliotis*^24^. Initially, the sequenced transcriptomes from two sequencers (Illumina (313.8 GB) and IsoSeq (27.7 GB)) were mapped to the *O. fasciatus* repeat-masked draft genome using Tophat^25^, and the transcript/gene structural boundaries were predicted using Cufflink^25^ and PASA^26^. To train the *ab initio* gene modeler and the EVM (which includes Exonerate^27^, AUGUSTUS^28^, and GENEID^29^), several genomes (*Gasterosteus aculeatus, Oreochromis niloticus, Tetraodon nigroviridis, Takifugu rubripes, Oryzias latipes, Danio rerio,* and *Homo sapiens*) were used for prediction. Finally, the predicted gene and transcripts models from the EVM and *ab initio* modeler were subjected to the consensus gene modeler (which includes EVidenceModeler^30^) to produce the final gene and transcript models. Finally, the consensus transcripts were subjected to functional annotation from biological databases (NCBI - NR databases, Uniprot, Gene Ontologies and KEGG pathways) by using Blast2GO^31^ (Fig. 1b). From this annotation, 50% of the genes are highly similar to *Larimichthys crocea* (Fig. 2b).

### Code availability

Throughout this study, we were not used any custom specific codes. The command line at each step were executed as instructed in the respective bioinformatics methods.

## Data Records

The entire data set used for draft assembly and its corresponding functional and structural annotations were deposited in public repositories. The DNA sequence libraries were deposited in NCBI (Data Citation 1) and see Table 1 for the details. The final assembly super-scaffold were submitted to NCBI Assembly (Data Citation 2) and see Table 2 for details. Moreover, the other files, such as the assembled contigs, scaffolds, and annotation tables, were stored in figshare (Data Citation 3) and see Table 4 for the details.

## Technical Validation

Throughout this study, every step was validated with the given metrics. The sampled fish were cultured under controlled conditions in the FRRI. Furthermore, the sequence libraries were quantified with different parameters. For Illumina, the isolated DNA spectrophotometer ratios (SP) were 260/280 ≥ 1.6 and total DNA ≥ 1.1 μg with minimum 20 ng/μl, and for PacBio, the SP was 260/280 ≥ 1.6 and 260/230 ≥ 2.0 and total DNA ≥ 15 μg with minimum 200 ng/μl. Moreover, the default parameters were used in the bioinformatics methods.

## Additional information

**How to cite this article**: Shin, Y. *et al*. First draft genome sequence of the rock bream in the family *Oplegnathidae*. *Sci. Data*. 5:180234 doi: 10.1038/sdata.2018.234 (2018).

**Publisher’s note**: Springer Nature remains neutral with regard to jurisdictional claims in published maps and institutional affiliations.

## Supplementary Material



## Figures and Tables

**Table 1:** Summary of the complete sequence libraries used in this study.

S. No	Sample Type	Library type	Platform	Insert size (bp)/cell	Read length (bp)	Total length(Gb)	Coverage (X)	Preprocessed	Coverage (X)	SRA Accesion Number
1	DNA	Paired-end	Illumina-HiSeq2000	350	101	56.4	73.6	39.9	52.1	SRR5860988
2	DNA	Paired-end	Illumina-HiSeq2000	550	101	53.6	69.9	35.8	46.7	SRR5860989
3	DNA	Mate-pair	Illumina-HiSeq2000	3,000	101	31.1	40.6	16.2	21.1	SRR5860986
4	DNA	Mate-pair	Illumina-HiSeq2000	5,000	101	27.6	36.0	12.1	15.8	SRR5860987
5	DNA	Mate-pair	Illumina-HiSeq2000	8,000	101	26.2	34.2	2.4	3.1	SRR5860984
6	DNA	Mate-pair	Illumina-HiSeq2000	10,000	101	29.5	38.5	3.2	4.2	SRR5860985
7	DNA	Long Fragments	PacBio RSII	20 Kb	Max: 50,375/Min: 50			11.5	15.0	SRR5860983

**Table 2:** *Oplegnathus fasciatus* genome *de novo* assemblies.

Description	1^st^ scaffolding (w/o PacBio)	2^nd^ scaffolding (w/PacBio)
No. of scaffolds	31,533	4,149
No. of bases (bp)	762,490,804	766,301,214
Scaffold N50 (bp)	874,256	1,126,915
Maximum length (bp)	5,005,633	7,250,909
Minimum length (bp)	143	1,000
N (%)	5.3	5.2
No. of contigs	108,639	
No. of bases (bp)	730,022,001	
Contig N50 (bp)	37,752	
Minimum length (bp)	200	
Maximum length (bp)	462,101	
N (%)	0.5	

**Table 3:** Repeat elements present in the *Oplegnathus fasciatus* genome.

Categories	Subcategories	No. of Elements	Length Occupied	% of Sequences
SINEs		16,852	2,167,823	0.28
	MIRs	2,753	4,18,120	0.05
LINEs		76,644	19,492,079	2.54
	LINE1	1,232	5,34,505	0.07
	LINE2	31,556	7,363,574	0.96
	L3/CR1	149	53,174	0.01
LTR elements		10,054	2,940,460	0.38
	ERV_Class I	184	111,018	0.01
DNA elements		253,296	50,393,060	6.58
	hAT-Charlie	11,297	2,077,564	0.27
Unclassified		469,919	88,403,276	11.54
Total Interspersed repeats		163,396,698		21.32
Small RNA		5,689	758,706	0.1
Satellites		1,693	165,759	0.02
Simple repeats		334,581	14,726,054	1.92
Low complexity		41,697	2,428,206	0.32

**Table 4:** Datasets for this project submitted to the figshare repository and its data descriptions.

**File name (Assembly Files)**	**File type**	**Data description**
**Contigs/scaffolds/Super scaffold**		
Oplegnathusfasciatus_contig.fa	fasta	Genome assembly result file (CLC Assembly Cell)
Oplegnathusfasciatus_scaffold.fa	fasta	Genome assembly result file (SSPACE - scaffolding with Illumina MP reads)
Oplegnathusfasciatus_super_scaffold.fa	fasta	Genome assembly results file (SSPACE - scaffolding with PacBio long reads)
**Repeat (Masked and unmasked results)**		
Oplegnathusfasciatus_super_scaffold.fa.out	txt	Repeat annotation file by Repeat Masker
Oplegnathusfasciatus_super_scaffold.fa.tbl	txt	The summary file
Oplegnathusfasciatus_super_scaffold.fa.masked	fasta	Repeat masked genome assembly file
**Gene models (Gene Prediction Files)**		
Oplegnathusfasciatus_cds.fna	fasta	Predicted coding sequence
Oplegnathusfasciatus_gene.gff3	gff3	Annotated coding sequence, gff3 format file
Oplegnathusfasciatus_protein.faa	fasta	Predicted protein sequence
**Function annotation (Blast2go Files)**		
Oplegnathusfasciatus_gene_definition.xls	xls	Give the blast description table from blast2go files
Oplegnathusfasciatus_Interpro.xls	xls	InterPro database annotation table
Oplegnathusfasciatus_gene_KEGG.xls	xls	KEGG database annotation
Oplegnathusfasciatus_GO_annotation.tar	tar	Gene Ontologies (BP, MF. CC)
